# Relation of Erythema Nodisum to Tuberculosis

**Published:** 1915-06

**Authors:** 


**Relation of Erythema Nodisum to Tuberculosis.** Landouzy, Paris, Med. Press & Circular, cites a case in a girl of 27, with negative results of blood cultures and injection of 10 c.c. of blood into a guinea pig, but showing tubercle bacilli in sections of an excise nodule. The general condition, he terms tubercular septicaemia.

